# Association between demographic factors and mental health outcomes among primary and secondary school teachers in Southeast of China: A cross-sectional study

**DOI:** 10.1186/s40359-025-03769-8

**Published:** 2025-12-02

**Authors:** Suhong Xiao, Chen Wang, Lili Zhang, Xinyu Xiang, Ruth E. Cortright, Rishi S. Pednekar, Isabella Overby, Jaspal S. Mahal, Feifei Wang

**Affiliations:** 1 Department of Neurosurgery, Zhejiang Rongjun Hospital, Jiaxing City, 314000 People’s Republic of China; 2 Department of Nursing, School of Life and Health Sciences, Huzhou College, Huzhou, 313000 China; 3 Ninghua Second Experimental Primary School, Ninghua County, Sanming City, 365499 Fujian Province China; 4 Department of Health, Human Function, and Rehabilitation Sciences, District of Columbia, The George Washington University, Washington, 20052 USA; 5 Faculty of Nursing, College of Medicine, Jiaxing University, Jiaxing, 314001 People’s Republic of China

**Keywords:** Mental health, School teacher, Anxiety, Pressure, Education psychology

## Abstract

**Objective:**

This study aimed to investigate the mental health status of primary and secondary school teachers. This study sought to comprehensively assess the psychological symptoms and identify potential problems.

**Methods:**

This is a cross-sectional study. A total of 2711 (response rate: 84.72%) primary and secondary school teachers were recruited through a random sampling method from urban and rural areas. The Symptom Checlist-90 (SCL-90) was used to assess their mental status. Data were analyzed using statistical methods such as descriptive statistics, t - tests, and one-way ANOVA to explore differences in mental health status among different demographic groups.

**Results:**

In total, 7% of the participants showed mild to severe level of psychological distress. Specifically, symptoms of obsessive - compulsive (M ± SD: 1.72 ± 0.63) and depression (M ± SD: 1.56 ± 0.65) were relatively common. Statistically significant differences were found in mental health scores among teachers with different teaching experience, urbanicity, age and gender (*p* < .0001). Teachers with more than 10 years of teaching experience had higher psychological distress. Male teachers reported more severe symptoms compared with female teachers. Teachers teaching in urban areas faced with worse mental health than teachers teaching in rural areas.

**Conclusions:**

The study revealed that primary and secondary school teachers face certain mental health challenges. These findings emphasize the urgent need for educational institutions and relevant departments to pay attention to teachers’ mental health, develop comprehensive mental health promotion programs, and provide appropriate support based on different teachers’ characteristics to improve their mental well-being.

## Introduction

Teaching at primary and secondary schools is recognized as a highly stressful profession [1]. Chinese primary and secondary schools are under highly competitive educational environment, driven by the pursuit of good school performance [2]. Driven by the consistent demand for education quality control and reform, this education atmosphere not only affects students’ physical and mental health but also has a profound impact on the teaching environment, increasing the workload and pressure on teachers [3].​ Approximately 40% teachers leaves the profession after 5 years due to stress and burnout [4]. Primary and secondary school teachers play a pivotal role in education system, and their mental well-being has become a matter of significant concern across society.

Profession stress leads to emotional exhaustion, which impacts teacher’s teaching performance and education quality. Evidence increasingly shows that teachers face a higher risk of mental disorders and work-related stress compared with those in other professions [5]. Primary and secondary school teachers are exposed to greater job stress and are more prone to severe psychological problems [6, 7]. Significant relationships were found among education performance, workplace stress and burnout indicators [8]. A theoretical framework posits that high work demands (e.g., workload, pressure from reforms) can lead to chronic psychological strain and impaired health [9]. The Chinese educational context, with its intense performance pressure, presents a specific case of high work demands, making it a critical setting for mental health investigation. Primary and secondary school educators are confronted with the transformation of China’s education system from traditional knowledge-imparting to quality-oriented education. Teachers are not only tasked with fulfilling the country’s requirements for cultivating constructive talents but also bear the burden of redesigning teaching content and mastering technical tools, which leads to extra occupational stress [10].

The mental health of primary and secondary school teachers is of crucial importance. Poor mental health among teachers can not only affect their teaching effectiveness but also have a negative impact on students’ learning experiences and mental well-being [11, 12]. For instance, teacher’s well-being is positively associated with student’s well-being and negatively associated with psychological distress [12]. It is possible that feelings of distress may influence teacher’s capacity to maintain positive classroom environments and respond effectively to support them [13]. Mental health distress in school settings is often categorized into internalizing (e.g., depression, anxiety, and somatic complaints) and externalizing symptoms (e.g., irritability, anger, and interpersonal conflict) [14]. Understanding both dimensions provides a more comprehensive picture of teacher psychological well-being. Therefore, exploring the mental health status of primary and secondary school teachers is beneficial for the teachers themselves and essential for the healthy development of students and the improvement of the overall education quality​.

Previous research has established links between certain demographic factors and teacher mental health. For example, female teachers often report higher levels of such as anxiety and depression [15], while younger and less experienced teachers may be more vulnerable to burnout [16]. However, findings regarding factors such as education level, place of work (urban vs. rural), and marital status are less consistent within the high-pressure context of Chinese primary and secondary education [3, 17], indicating a significant research gap. A more nuanced investigation is needed to clarify how these demographics interact with the unique stressors of this environment.

This study has two primary objectives: (1) to quantitatively assess primary and secondary school teachers’ mental health status, including both internalizing and externalizing symptoms, and (2) to examine the relationships between specific sociodemographic factors (gender, age, education level, place of work, and marital status) and these mental health outcomes. We propose the following hypotheses:1) Teachers will report clinically significant levels of both internalizing and externalizing mental health symptoms. 2)Female teachers will report significantly higher levels of internalizing symptoms than male teachers. 3)Younger teachers will report higher levels of overall psychological distress compared to older teachers. 4) Significant differences in mental health symptoms will exist based on place of work (urban vs. rural), education level, and marital status.

This study highlights the importance of considering and addressing the mental health needs of teachers in school settings. The results of this study can serve as a basis for formulating strategies to prevent and intervene in teacher mental health issues.

## Methods

### Study design and participants

This study employed a cross-sectional survey design. G*Power software (version 3.1.9.7) was used to determine the minimum required sample size, which yielded a minimum sample of 1,259 participants. To ensure robust subgroup analyses across different demographics, we recruited a larger convenience sample of 2,711 primary and secondary school teachers from Sanming City, China.

### Sampling strategy and Inclusion/Exclusion criteria

A convenience sampling strategy was used for participant recruitment. With the approval of the local government, the survey was distributed to primary and secondary schools in Sanming City. The inclusion criteria were: (1) a certified full-time teacher employed at a primary or secondary school in Sanming City, and (2) at least one year of teaching experience. The exclusion criterion was being a part-time or substitute teacher.

### Measures

A self-developed questionnaire collected key demographic and professional characteristics, including gender (male, female), age, education level, place of work (primary school or secondary school), and marital status.

### Symptom checlist-90 (SCL-90)

The Symptom Checklist 90 (SCL-90) [18], is a self-report measurement for measuring psychological and psychiatric symptoms. The Chinese version of the SCL-90 has been extensively validated and demonstrates good psychometric properties within the Chinese population. the SCL-90 demonstrated excellent reliability and validity [19]. It serves as an effective tool for assessing the psychological and mental well-being by self-assessment, enabling measurement of the prevalence and severity of psychological disorders, mental illnesses, and mental disturbances. The SCL-90 consists of 90 items that assess ten dimensions of psychological condition, nine dimensions are psychological outcomes with one additional dimension focusing on sleep and eating habits. Each item is rated on a scale from 0 to 4, reflecting the intensity of an individual’s feelings during the past week [20]. Subsequently, scores ranging from 1 to 5 are assigned based on these ratings.

Psychological distress level is rated between “none” and “severe”. A total score ≥ 160 is considered indicative of significant psychological distress. When the mean SCL-90 score is within the range of 1 to 1.5, it suggests that the participants have no perception of the symptoms enumerated in the scale. A score between 1.5 and 2.5 indicates that the participants experience a certain degree of symptoms, yet these symptoms do not manifest frequently. If the score falls between 2.5 and 3.5, it implies that the participants are symptomatic, with the severity level being mild to moderate. When the score is in the range of 3.5 to 4.5, it denotes that the participants are symptomatic, and the severity level is moderate to severe. A score between 4.5 and 5 signifies that the participants are symptomatic, and both the frequency and intensity of the symptoms are extremely high.

### Data collection process

Data were collected online via the “Questionnaire Star” platform (https://www.wjx.cn) from April 2024 to May 2025. A sharable QR code linking to the questionnaire was distributed to school administrators for dissemination among teaching staff. The first page of the online survey displayed the informed consent form, detailing the study’s purpose, confidentiality, and the voluntary nature of participation. Proceeding to the questionnaire after reading this information required participants to click “YES,” indicating their electronic consent. All collected data were anonymized and stored securely on the platform, accessible only to the research team for analysis. A piloted study with 6 teachers were conducted to check for clarity and wording to the final survey.

### Statistical analyses

The SCL-90 total score and its subscale scores were expressed as mean ± standard deviation (M ± SD). Categorical data were expressed as percentages (%). Differences in SCL-90 scores among the participants with demographic characteristics were analyzed using independent t-tests or one-way analysis of variance (ANOVA). The Wilcoxon test was used to analyze the gender difference in the mental status of the participants. Statistical analyses were performed using SAS (version 9.4, Cary, NC, USA) and RStudio (version 2024.12.1 + 563, 2025 posit software, PBC). A two-tailed significance test was used and the level of statistical significance was set at *p* <.05.

## Results

### Characteristics of participants

The present study included 2711 primary and secondary teachers, with 1630 (60.13%) female teachers. In summary, 2074 (76.5%) teachers were above 30 years old, and about similar number of teachers (2063, 76.10%) were under graduated. In addition, the majority of teachers (2245, 82.81%) were married. Furthermore, the results showed that more than half of the teachers worked more than 10 years (1667, 61.49%), and more teachers were living in urban (1563, 57.65%) than in rural areas (1148, 42.35%). There were significant differences in the SCL-90 scores regarding age, gender, years of working experience and work place (*p* <.0001). There were no significant differences in education level and marital status (*p* >.05). Table 1 summarizes the socio-demographic characteristics of the participants.

**Table 1:** Characteristics of participants by SCL-90 score

Characteristics	*N* (%)	SCL-90 score (M ± SD)	F value	Effect size	*p*
Gender			1.41	0.21	< 0.0001
Male	1081(39.87)	1.57(0.63)			
Female	1630(60.13)	1.45(0.53)			
Age (years)			9.92	0.04	< 0.0001
Less than 30 years	637(23.50)	1.37(0.48)			
31–40 years	581(21.43)	1.40(0.49)			
41–50 years	735(27.11)	1.53(0.60)			
Above 50 years	758(27.96)	1.64(0.64)			
Education			1.48	0.0002	0.23
Non-tertiary education	635(23.42)	1.48(0.60)			
Undergraduate education	2063(76.10)	1.50(0.56)			
Postgraduate education	13(0.48)	1.53(0.80)			
Marital status			1.66	0.006	0.19
Single	410(15.12)	1.40(0.51)			
Married	2245(82.81)	1.51(0.58)			
Divorced	56(2.07)	1.63(0.62)			
Work (years)			11.15	0.034	< 0.0001
Less than 5 years	685(25.27)	1.34(0.47)			
5–10 years	359(13.24)	1.42(0.51)			
More than 10 years	1667(61.49)	1.58(0.61)			
Workplace			1.33	0.19	< 0.0001
Urban	1563(57.65)	1.44(0.02)			
Rural	1148(42.35)	1.54(0.02)			

Abbreviations: *SCL-90* symptom checklist-90, *SD* standard deviation

### Mental health status

**Fig. 1:**
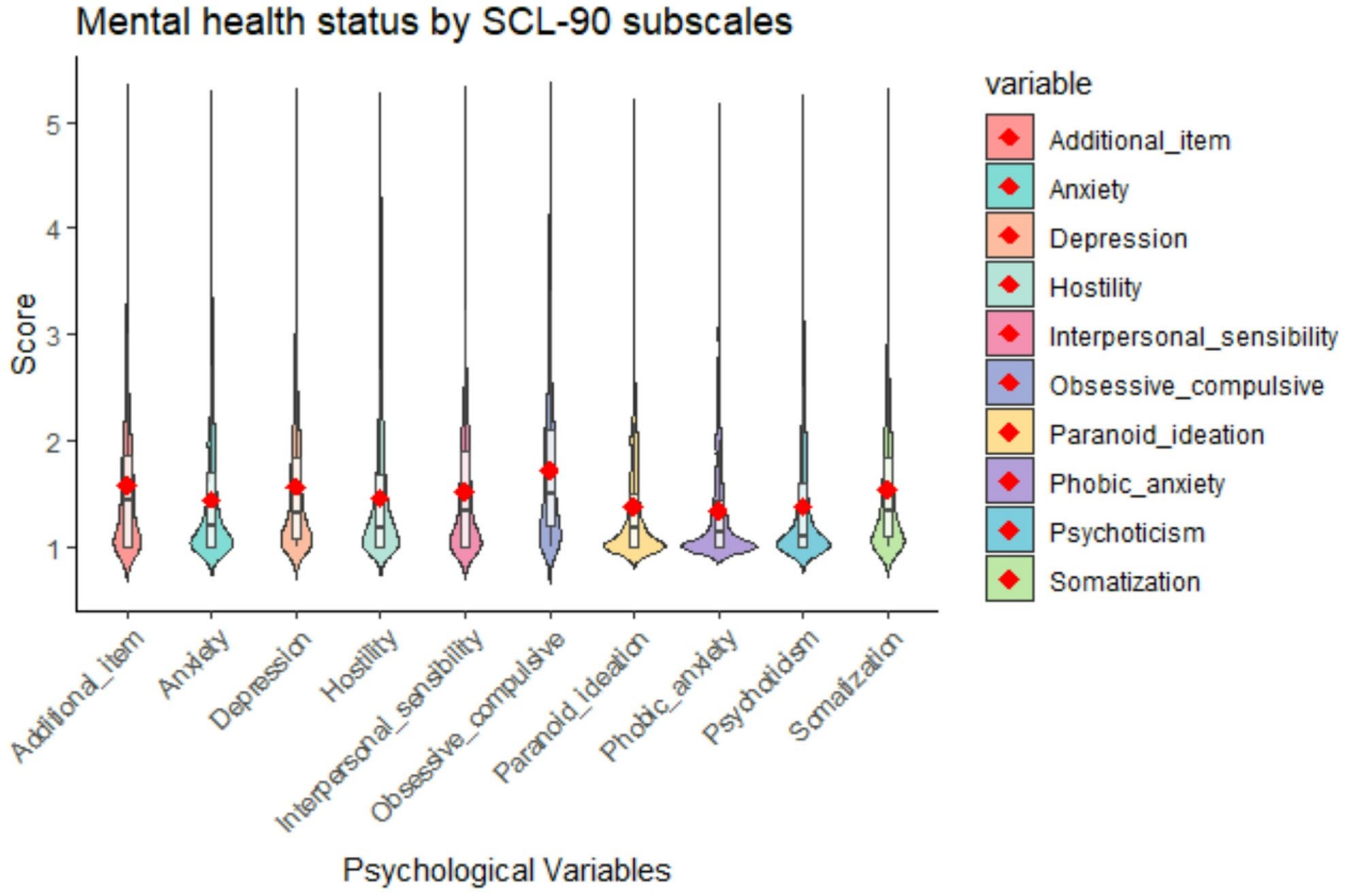
Mental health status by SCL-90 subscales among primary and secondary school teachers

Among the 2711 participants, 36.85% (999/2711) were detected with mild to severe mental health problems. Ten mental health problems (i.e., somatization, obsessive compulsive, interpersonal sensitivity, depression, anxiety, hostility, phobic anxiety, paranoid ideation, psychoticism, additional item) were summarized (see Table 2). The distributions of these SCL-90 subscales among primary and secondary school teachers are shown in Fig. 1. Teachers’ mental status was poor (Mean = 1.50, SD = 0.57), especially in obsessive compulsive (1226, 45.22%). 2067(76.24%) participants were free of phobic anxiety, and no one was diagnosed with phobic anxiety (Mean = 1.33, SD = 0.52).

**Table 2:** Prevalence of mental health by SCL-90 subscales

Subscales	Severity of mental health *N* (%)					Mean (SD)
	None	Mild	Mild to moderate	Moderate to severe	Severe	
SCL_total	1712(63.15)	809(29.84)	160(5.90)	28(1.03)	2(0.07)	1.50(0.57)
Somatization	1586(58.50)	883(32.57)	192(7.08)	45(1.66)	5(0.18)	1.54(0.63)
Obsessive compulsive	1226(45.22)	1128(41.61)	277(10.22)	70(2.58)	10(0.37)	1.72(0.71)
Interpersonal sensitivity	1629(60.09)	878(32.39)	172(6.34)	29(1.07)	3(0.11)	1.52(0.61)
Depression	1591(58.69)	879(32.42)	185(6.82)	52(1.92)	4(0.15)	1.56(0.65)
Anxiety	1803(66.51)	719(26.52)	145(5.35)	39(1.44)	5(0.18)	1.44(0.60)
Hostility	1730(63.81)	793(29.25)	149(5.50)	34(1.25)	5(0.18)	1.45(0.58)
Phobic anxiety	2067(76.24)	519(19.14)	112(4.13)	12(0.44)	0(0.04)	1.33(0.52)
Paranoid ideation	1863(68.72)	699(25.78)	117(4.32)	29(1.07)	3(0.11)	1.37(0.55)
Psychoticism	1911(70.49)	654(24.12)	122(4.50)	20(0.74)	4(0.15)	1.37(0.54)
Additional item	1531(56.47)	918(33.86)	218(8.04)	40(1.48)	4(0.15)	1.57(0.64)

*SCL-90* Symtom Checlist-90, *SD* standard deviation

### Subgroup analyses of mental health

**Fig. 2:**
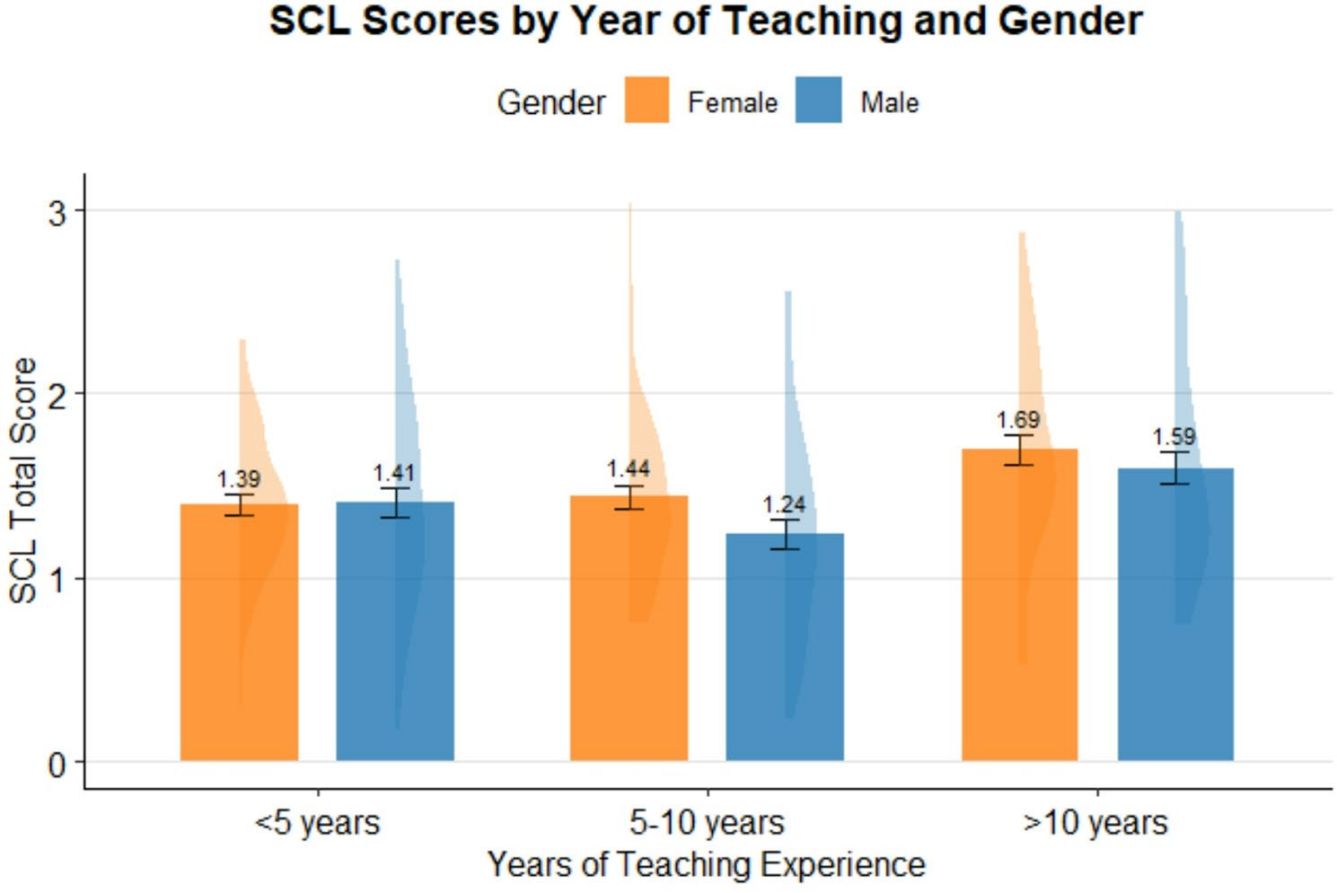
Difference of mental health with year of teaching experience

Further subgroup analyses were conducted to classify the associations of demographic characteristics and subscales of mental health. The results showed significant differences in mental health subscales regarding gender and urbanicity (*p* <.0001). In general, male teachers faced with lower mental health compared with female teachers (see Table 3). In addition, teachers living in urban areas are under worse mental health than teachers living in rural areas (see Table 4). Moreover, mental health score between male and female teachers was compared by year of teaching experience. Teachers working for more than 10 years experienced worse mental health compared with those who worked shorter (see Fig. 2).

**Table 3:** Comparisons of SCL-90 subscales by sex

Subscales	Gender			
	Female M(SD)	Male M(SD)	F value	*p*
Somatization	1.49(0.59)	1.60(0.69)	1.37	< 0.0001
Obsessive compulsive	1.67(0.67)	1.79(0.76)	1.28	< 0.0001
Interpersonal sensitivity	1.46(0.56)	1.60(0.66)	1.41	< 0.0001
Depression	1.51(0.61)	1.63(0.69)	1.31	< 0.0001
Anxiety	1.40(0.56)	1.50(0.65)	1.34	< 0.0001
Hostility	1.42(0.54)	1.50(0.63)	1.35	< 0.0001
Phobic anxiety	1.31(0.49)	1.37(0.57)	1.37	< 0.0001
Paranoid ideation	1.32(0.49)	1.46(0.62)	1.60	< 0.0001
Psychoticism	1.32(0.49)	1.45(0.61)	1.58	< 0.0001
Additional item	1.50(0.59)	1.68(0.71)	1.45	< 0.0001

*M* mean, *SD* standard deviation

**Table 4:** Comparisons of SCL-90 subscales by urbanicity

Subscales	Work place			
	Urban M(SD)	Rural M(SD)	F value	*p*
Somatization	1.58(0.66)	1.48(0.59)	1.28	< 0.0001
Obsessive compulsive	1.78(0.73)	1.64(0.66)	1.22	< 0.0001
Interpersonal sensitivity	1.55(0.63)	1.46(0.56)	1.26	< 0.0001
Depression	1.62(0.68)	1.48(0.59)	1.33	< 0.0001
Anxiety	1.49(0.65)	1.37(0.53)	1.49	< 0.0001
Hostility	1.50(0.61)	1.39(0.52)	1.40	< 0.0001
Phobic anxiety	1.36(0.55)	1.29(0.48)	1.28	< 0.0001
Paranoid ideation	1.41(0.58)	1.32(0.51)	1.30	< 0.0001
Psychoticism	1.41(0.57)	1.32(0.49)	1.35	< 0.0001
Additional item	1.61(0.67)	1.52(0.59)	1.29	< 0.0001

*M* mean, *SD* standard deviation

## Discussion

The current study explored mental health related sociodemographic factors and comprehensively analyzed their relationship among primary and secondary school teachers in southeast SS of China. This study specifically investigated the unique psychological conflicts generated by sociodemographic among primary and secondary school teachers in rural areas of China. Primary and secondary school teachers are frequently regarded as the gardeners of education, and their mental health may significantly impact their teaching performance and education quality [21]. Compared to the national norm [3], the mental health status of the teachers was poor, especially for somatization, interpersonal sensitivity, depression, anxiety, hostility, phobic anxiety, and psychoticism. Our results also indicated that age, gender, urbanicity, and year of teaching experience significantly influence mental health outcomes.

Our findings align with existing literature showing that socio-demographic factors such as gender, age and year of teaching experiences significantly influence teachers’ mental health. The most reported correlates in previous studies were sex, age, gender, marital status, job satisfaction, subject taught and years of teaching, which may correlate with depression, anxiety and stress among teachers [6]. Other factors such as climate and social support may also relate to depression and anxiety [22]. Regarding gender, sex correlates with stress although there are some conflicting reports. For instance, some studies report that female teachers experience more stress than their male counterparts [23, 24]. However, this may be context-dependent, for example, no sex difference was reported among teachers with different education levels [25]. Conversely, male teachers who have additional demand for supporting family, and trying to accomplish both roles at home and work, may be a source of mental distress [26]. Furthermore, among the primary and secondary school teachers, work-family conflict has been reported to be significantly associated with stress and anxiety [27, 28].

Year of working experience is associated with teachers’ mental health. The Chinese version of the SCL-90 has been previously employed to assess various aspects of participants’ mental health, with one study indicating that most symptoms of psychological distress increased as teaching experience increased [29]. This can be explained by the fact that the primary and secondary school teachers’ job is an emotional effort, and teachers have to feel, understand, and react to students’ emotions appropriately [30]. For instance, a study by Harding et al. [12] found that teachers experiencing psychological distress reported decreased productivity and increased difficulty with classroom management [31]. Thus, given the emotional nature of elementary teaching, year of teaching is associated with teachers’ mental health symptoms [32]. Emotional exhaustion, detachment from work, and feelings of personal or professional inadequacy as well as reduced productivity have been reported as reasons for job burnout, which significantly predicted depressive symptoms [33].

In China, teaching can be a high-pressure profession, where teachers are responsible for lesson design, assignments, classroom performance, and communicating with parents. In recent years, China’s education reform has undergone several policies. One of the most important policies is “Double-Reduce Policy 2021” in compulsory education, which was launched to reduce students’ burden of learning. The “Double Reduction” policy, aimed at alleviating the academic burden on students, imposes specific restrictions on teachers—such as prohibiting excessive homework assignments and disallowing extracurricular tutoring [34]. However, research has found that it was difficult to fundamentally decrease teachers’ occupational anxiety caused by the policy [35]. The mental health of Chinese primary and secondary teachers (with 36.85% showing mild to severe mental health problems) is poorer compared with teachers from Chile (28.6% with mental health problems) [36]. Moreover, there is a trend that Chinese teachers’ mental health problems have increased compared with previous studies [37].

While the socio-educational context of China is unique, valuable insights may be drawn from established support systems in other nations. For instance, countries like Finland and Singapore, which also maintain high academic standards, implement comprehensive strategies such as mandated protected non-instructional time, access to school-based psychological consultants, and distributed leadership models that empower teachers. It is imperative to adapt these international models with sensitivity to China’s distinct socio-educational landscape, including regional inequities, examination-oriented culture, and centralized policy implementation [38, 39]. Thus, a support framework for mental health with Chinese characteristics, tailored for primary and secondary school educators may enhance their overall well-being and the quality of national education quality.

### Limitations

There are several limitations to our study. First of all, the data collected in this study were self-reported, which could lead to methodological biases. Second of all, the participants may have concealed their true psychological status due to fear of leaking private information and the reputation of the school. Third of all, the data collection followed a clustered sampling method, which may limit generalization of the results. Fourth of all, the possible affected stressors (e.g. economic status, social issues) were not investigated. Finally, this study was conducted in a less-developed area of China, which may bias the results.

## Conclusions

This study highlights the necessity for psychological improvement among primary and secondary school teachers, underscoring the urgent need for holistic mental health interventions. The findings align with global evidence on educator wellbeing while revealing context-specific vulnerabilities. Education institutions may enhance teacher well-being by fostering a supportive and empathetic culture, providing gender-specific resources, and encouraging healthy work environment. Future studies are expected to employ longitudinal designs to examine causal pathways, and to explore the development, implementation, monitoring, and evaluation of intervention programs for improving mental health outcomes among teachers. Prioritizing teacher mental health is not merely an occupational concern but a fundamental prerequisite for sustaining educational quality and student development. In addition, governments, school boards and policymakers need to collaborate together on the design and implementation of measures to enhance teachers’ mental health, teaching performance and quality of life.

## Data Availability

Data available on request from the corresponding author FW.
